# Contribution of Human FcγRs to Disease with Evidence from Human Polymorphisms and Transgenic Animal Studies

**DOI:** 10.3389/fimmu.2014.00254

**Published:** 2014-05-30

**Authors:** Caitlin Gillis, Aurélie Gouel-Chéron, Friederike Jönsson, Pierre Bruhns

**Affiliations:** ^1^Laboratoire Anticorps en Thérapie et Pathologie, Département d’Immunologie, Institut Pasteur, Paris, France; ^2^U760, INSERM, Paris, France; ^3^Department of Anesthesia and Intensive Care, Hospital of Bichat-Claude Bernard, Public Assistance-Hospitals of Paris, Paris, France

**Keywords:** IgG receptors, transgenic mice, anaphylaxis, autoimmune diseases, genetic polymorphisms and disease association, human IgG receptors

## Abstract

The biological activities of human IgG antibodies predominantly rely on a family of receptors for the Fc portion of IgG, FcγRs: FcγRI, FcγRIIA, FcγRIIB, FcγRIIC, FcγRIIIA, FcγRIIIB, FcRL5, FcRn, and TRIM21. All FcγRs bind IgG at the cell surface, except FcRn and TRIM21 that bind IgG once internalized. The affinity of FcγRs for IgG is determined by polymorphisms of human FcγRs and ranges from 2 × 10^4^ to 8 × 10^7^ M^−1^. The biological functions of FcγRs extend from cellular activation or inhibition, IgG-internalization/endocytosis/phagocytosis to IgG transport and recycling. This review focuses on human FcγRs and intends to present an overview of the current understanding of how these receptors may contribute to various pathologies. It will define FcγRs and their polymorphic variants, their affinity for human IgG subclasses, and review the associations found between FcγR polymorphisms and human pathologies. It will also describe the human FcγR-transgenic mice that have been used to study the role of these receptors in autoimmune, inflammatory, and allergic disease models.

## Introduction on Human FcγRs: Definition and Basic Functions

Human myeloid cells, NK cells, and B cells are equipped with a variety of receptors that enable their interaction with monomeric or aggregated immunoglobulins, antigen–antibody immune complexes, and opsonized (antibody-coated) particles, cells, or surfaces. Most of these receptors bind the Fc portion of immunoglobulins (receptors for the Fc portion of immunoglobulins, FcR) and endow these cells with the capacity to interact with IgM, IgA, IgG, and/or IgE. This review will focus on IgG-binding human FcRs, FcγRs.

**Figure 1:**
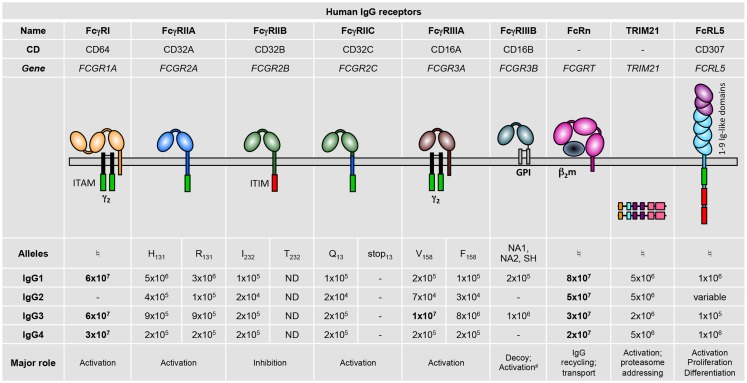
**Human IgG receptor family**. Alleles are identified by the amino acid variant in the protein (e.g., H_131_), or by the name of the allelic variants (NA1, NA2, or SH). Binding affinities for the various immunoglobulin subclasses are given as M^−1^. High-affinity interactions are indicated in bold. –, no binding; ND, not determined; ^♮^No allelic variants have yet been described that affect binding affinity. ^#^Associates with integrins. ITAM, immunoreceptor tyrosine-based activation motif; γ_2_, dimer of FcRγ subunits; ITIM, immunoreceptor tyrosine-based inhibitory motif; GPI, glycosyl-phosphatidylinositol; β_2_m, β_2_-microglobulin.

Humans express nine FcγRs: the six classical FcγRs, FcγRI, FcγRIIA, FcγRIIB, FcγRIIC, FcγRIIIA, and FcγRIIIB; as well as FcRn, FcRL5 (1, 2), and TRIM21 (3) (Figure 1). These FcγRs all bind IgG on the surface of the cells expressing them, except FcRn (4, 5) and TRIM21 (6, 7) that bind IgG once internalized. Notably, all IgG receptors bind at least two human IgG subclasses, albeit with varying binding affinity: the association constants (K_A_) of IgG–FcγR interactions range from 8 × 10^7^ down to 2 × 10^4^ M^−1^ (8) (Figure 1). Historically, FcγRs were categorized as either *low-affinity* receptors that can only bind IgG when present in an immune complex, aggregated, or opsonized; or *high-affinity* receptors that can also bind free or monomeric IgG. This terminology has become rather obsolete considering reports of high- and low-affinity interactions for a single receptor toward different Ig subclasses. Furthermore, although the prevailing belief was that occupancy of high-affinity receptors with pre-bound monomeric IgG prevents their participation in immediate IgG-dependent reactions; this has recently been refuted *in vivo* (9). Adding to this complexity, human FcγR polymorphisms that modulate affinity for some human IgG subclasses have been described (8) (refer to part 2; Figure 1).

**Figure 2:**
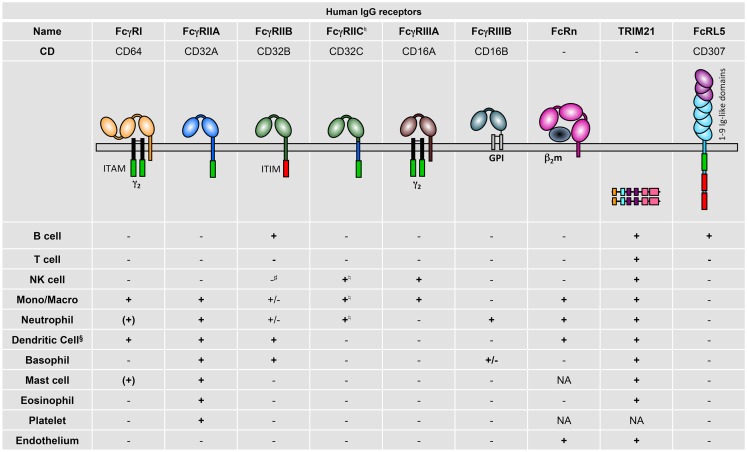
**Human IgG receptor expression pattern**. + indicates expression; (+), inducible expression; ±, very low percentages or rare subsets express the receptor; −, no expression; and NA, not analyzed; Mono/Macro, monocytes, and/or macrophages. ^§^Refer to the review by Guilliams et al. for specific expression on human DC subtypes (20). ^♮^In Fcgr2c-ORF persons (17). ^#^Detectable and functional expression in non-conventional Fcgr2c-Stop persons (17).

Human FcγR expression on different cell types has been fairly comprehensively described, mostly by the use of FcγR-specific monoclonal antibodies (mAb) but also from data using mRNA profiling (Figure 2). Generally, the following observations can be made: hFcγRI (CD64) is restricted to monocytes/macrophages and dendritic cells and is inducibly expressed on neutrophils (10) and mast cells (11); hFcγRIIA (CD32A) is expressed on all myeloid cells but not on lymphocytes; hFcγRIIB (CD32B) is expressed at high levels only on B cells (12) and basophils (13). It is also expressed on tissue macrophages and dendritic cells (12), but only at low levels on 20% of circulating monocytes and 4% of circulating neutrophils (12, 14), and is not expressed on primary skin mast cells (15); hFcγRIIC (CD32C; refer to Section “Human FcγR Polymorphisms” for its “stop_13_” polymorphism) is expressed on NK cells (16), monocytes, and neutrophils (17); hFcγRIIIA (CD16A) is expressed on NK cells and monocytes/macrophages; hFcγRIIIB (CD16B) is highly expressed on neutrophils and at low levels on some basophils (18). TRIM21 (aka Ro52) was described to be widely expressed among lymphoid and myeloid populations, but also on endothelial cells (19). FcRL5 has been reported to be restricted to B cells (2).

These expression patterns highlight that hFcγRIIA is the only activating IgG receptor constitutively expressed by mast cells, basophils, neutrophils, and eosinophils, and that FCRL5 is the only activating IgG receptor constitutively expressed by B cells. Importantly, signal transduction events induced by human activating IgG receptors may be negatively regulated by hFcγRIIB only in B cells, dendritic cells, and basophils, and rare fractions of monocytes and neutrophils. Indeed, mast cells, NK cells, and most neutrophils and monocytes do not express this inhibitory receptor. hFcRn has been reported in dendritic cells, monocytes/macrophages (21), neutrophils (22), and endothelial cells (23), but expression on platelets and mast cells has not been examined so far.

These patterns correspond to the expression of FcγRs in healthy individuals. These may be modified during pathological conditions or following therapeutic treatments. Certain cytokines for example have been reported to up-regulate or down-regulate some hFcγRs; e.g., B cells express higher levels of hFcγRIIB following IFN-γ but lower levels following IL-4 stimulation, whereas opposite effects have been reported for monocytes [reviewed in Ref. (24)]. On the latter cells, expression of hFcγRIIA is increased following IFN-γ and decreased following IL-4 stimulation (25). IL-3 stimulation, however, induces higher expression of both receptors (activating hFcγRIIA and inhibitory hFcγRIIB) on basophils (13). Mucosal mast cells express hFcγRI upon IFN-γ stimulation (11). Surprisingly, IL-3 stimulation of primary monocytes did not modify hFcγRI expression, but increased its ability to bind IgG-immune complexes and to induce intracellular activation signals (26).

Activating FcγRs signal through an immunoreceptor tyrosine-based activation motif (ITAM) that is either present in their intracytoplasmic domain or in associated signaling subunits, such as the FcRγ chain (Figure 1), the FcRβ chain (exclusively in mast cells and basophils), or the CD3ζ chain (exclusively in NK cells). These ITAM-containing structures allow FcγRs, once aggregated by multimeric ligands, to activate signaling cascades via SRC family kinases and spleen tyrosine kinase (SYK) leading to cell activation, cytokine/chemokine production, and cell migration (27–29). The inhibitory receptor FcγRIIB possesses instead an immunoreceptor tyrosine-based inhibition motif (ITIM) in its intracytoplasmic domain (30), which allows this receptor, once co-engaged with an activating FcγR, to recruit the inositol polyphosphate-5-phosphatase SHIP1 (31) that counteracts the signaling cascades initiated by activating FcγRs (24). FcRL5 possesses both an ITAM and two ITIMs; however, it has been reported to exert mainly negative regulatory functions (32). IgG receptors devoid of both ITAM and ITIM may induce cell activation by associating with other receptors at the cell membrane, for example the glycophosphatidylinositol-anchored FcγRIIIB (33, 34) associates with integrins (35); or by activating transcription pathways or proteasome-related mechanisms as does TRIM21 (7, 36).

Internalization of antibodies, and of the antigens they are bound to, represents the only shared function of IgG receptors expressed at the cell surface (that is, all except FcRn and TRIM21), whether ITAM-bearing, ITIM-bearing, or neither. FcγRs thereby enable antigen capture and internalization by all FcγR-expressing nucleated cells, as well as phagocytosis of opsonized bacteria, viruses, or cells by phagocytes. FcRn is the only receptor enabling transcytosis of IgG or IgG-IC by polarized cells (23). Enhanced uptake of antibody-bound antigen enables antigen-presenting cells to activate antigen-specific T cells considerably more efficiently than free antigen (37), signifying the pivotal role of FcγRs in the initial phase of humoral and cellular immune responses. Receptors that bind IgG only when it has already been internalized, FcRn (the topic of this review series) and the ubiquitously expressed intracellular receptor TRIM21, may possibly contribute to this phenomenon [reviewed in Ref. (20)].

## Human FcγR Polymorphisms

### Definitions

The multiplicity of human FcγRs (Figure 1) is increased by a series of genetic polymorphisms, for which we will describe herein only those leading to known functional modifications. These are summarized in Table 1.

**Table 1:** **Summary of human FcγR polymorphisms**.

Receptor	Variant	Effect	Reference
FcγRIIA	H/R_131_	H_131_: ***↗*** binding of IgG2 and IgG1	(8, 38–40)
		***↗***Immune complex-opsonization	
	FcγRIIA-exon 6*	***↗***Activation following IgG stimulation	(41)
FcγRIIB	−386G/c	***↗***promoter activity: thus, ***↗*** FcγRIIB expression	(24, 42)
	−120T/a	
	I/T_232_	T_232_: ***↘*** inhibitory function	(43)
FcγRIIC	Q/stop_13_	Q_13_: expression on NK cells, monocytes, neutrophils	(17)
		***↗***IgG-induced cell activation	
	CNV	Correlation with protein expression levels	(44)
FcγRIIIA	V/F_158_	V_158_: ***↗*** binding to IgG1, IgG2, IgG3	(8, 45, 46)
		***↗***Cell activation	
	CNV	Correlation with protein expression levels; impaired NK cell cytotoxic function	(47)
FcγRIIIB	NA1/NA2/SH	NA1: ***↗*** phagocytosis of IgG-immune complexes	(48–51)
		SH: ***↗*** FcγRIIIB expression levels	
	CNV	Correlation with protein expression levels	(52)

#### FcγRIIA

A polymorphism resulting in the presence of a histidine or an arginine residue at position 131 may also be referred to as low-responder (H_131_) or high-responder (R_131_) (38). The FcγRIIA-H_131_ allotype was originally reported to allow binding to IgG2 (53), subject to ethnic variation (54, 55), and was later described to also have increased binding for IgG3 (39). More recently, we have identified that only the binding to IgG1 and IgG2 are increased for H_131_ compared to R_131_ (8).

A novel splice variant of *FCGR2A*, FcγRIIA-exon 6*, containing an expressed cryptic exon 6* was identified in 2013 (41), and is associated with increased neutrophil sensitivity to IgG stimulation (56).

#### FcγRIIB

Single-nucleotide polymorphisms (SNPs) at positions 386 [IIB-386 (G/c)] and 120 [IIB-120 (T/a)], collectively constitute the 2B.4 promoter haplotype, which displays increased binding capacity for transcription factors GATA4 and Yin-Yang1, resulting in increased promoter activity and higher expression of FcγRIIB on monocytes, B lymphocytes, neutrophils, and myeloid DCs (24, 42).

A polymorphism encoding an isoleucine to threonine substitution at position 232 in the transmembrane domain of FcγRIIB (T_232_) may disable receptor function via exclusion from lipid rafts (43, 57).

#### FcγRIIC

In 20% of individuals *FCGR2C* encodes for a glutamine at position 13 (Q_13_ or ORF) and FcγRIIC is expressed; but in 80% of individuals a SNP generates a stop codon (stop_13_), in which case *FCGR2C* represents a pseudogene (16).

A subset of individuals carrying *FCGR2C-ORF* do not express FcγRIIC due to splice-site mutations and loss of exon 7. Inversely, this polymorphism leads to the expression of inhibitory hFcγRIIB expression on NK cells that has been shown to negatively regulate IgG-induced NK cell activation (17).

#### FcγRIIIA

A SNP determines the presence of a valine or phenylalanine at position 158 (45). The FcγRIIIA-V_158_ variant demonstrates increased affinity for IgG1, IgG2, and IgG3, and increased IgG-induced cell activation and elimination of immune complexes (8, 46, 58).

#### FcγRIIIB

FcγRIIIB bears the neutrophil antigen (NA) in its membrane-distal Ig-like domain, generating three variants termed NA1 (R_36_ N_65_ A_78_ D_82_ V_106_), NA2 (S_36_ S_65_ A_78_ N_82_ I_106_) (48, 59), and SH (S_36_ S_65_ D_78_ N_82_ I_106_) (50) that do not demonstrate detectable differences in affinity for hIgG subclasses (8). The NA1 allotype was, however, reported to increase phagocytosis of IgG-opsonized particles (49). The SH allotype has been associated with higher FcγRIIIB expression levels (51).

#### Gene copy number variation (CNV)

Recognized as an important indicator for inter-individual differences, can alter the expression of activating IgG receptors. The balance between activating and inhibitory FcγRs can therefore be perturbed, altering cellular responses toward IgG-immune complexes. CNV of *FCGR2C, FCGR3A*, and *FCGR3B* (Table 1) have been shown to correlate with protein expression levels. Duplications of the gene encoding *FCGR3B* can lead to the expression of the three different FcγRIIIB variants (NA1, NA2, and SH) in a single individual (51). CNV in *FCGR3A* (deletion of one allele) correlated with a reduced expression of FcγRIIIA on NK cells and impaired cytotoxic function (47). Deletion of a large portion of the *FCGR* locus, including *FCGR2C* and *FCGR3B*, also resulted in abnormal expression of FcγRIIB on NK cells, presumably due to deletion of upstream regulatory elements. Expression of this inhibitory receptor enabled negative regulation of IgG-induced NK cell activation (17). To the extent of our knowledge, CNV of the *FCGR2A* and *FCGR2B* genes have not been reported (47).

### Association with disease susceptibility and/or success of antibody-based therapies

Several *FCGR* polymorphisms modify the affinity between FcγRs and human IgG, and therefore the efficacy of immune complex clearance can be affected. Reduced immune complex clearance is indeed a risk factor for diseases like Systemic Lupus Erythematosus and Wegener’s granulomatosis (60, 61). Other polymorphisms may favor detrimental inflammatory responses and thus predispose to autoimmunity. Diseases that have been associated with FcγR polymorphisms are presented in Table 1.

FcγR polymorphisms may also influence patients’ response to treatment with intravenous immunoglobulin and therapeutic mAb. Almost all mAb used in therapy are based on human IgG1 antibodies, either chimeric mouse/human or fully human, allowing their interaction with all human FcγRs (8, 62). The first report to assess the predictive value of FcγR polymorphisms in responses to antibody therapies associated homozygous *FCGR3A*-V/V_158_ individuals with better clinical responses to anti-CD20 therapy (Rituximab) in the treatment of non-Hodgkin lymphomas (63). Homozygous *FCGR3A*-V/V_158_ individuals have since been found to have improved biological responses to anti-CD20 therapy in immune thrombocytopenia (64) and rheumatoid arthritis (RA) (65); and anti-TNF-α therapy (Infliximab) to treat Crohn’s disease (66, 67); compared to carriers of one or two *FCGR3A*-F_158_ alleles. In arthritis patients, however, findings are controversial regarding the association of *FCGR3A* polymorphisms with clinical response to TNF-α inhibitors (infliximab, adalimumab, etanercept): although one study describes a better clinical response in *FCGR3A*-F/F_158_ patients (68); another, larger study with a more homogenous patient cohort found no association (69). Homozygous *FCGR3A*-V/V_158_ individuals were more likely to experience complete remission from immune thrombocytopenia following medication, but conversely remission rates after splenectomy were higher in homozygous *FCGR3A*-F/F_158_ or heterozygous individuals (70). The *FCGR2A*-H131 variant associates with susceptibility to Kawasaki Disease (Table 1), whereas responsiveness to IVIG therapy in Kawasaki Disease patients is strongly associated with the *FCGR3B* genotype: the NA1 variant significantly decreases the odds of an appropriate clinical outcome (71). Similarly, CNV of both *FCGR3B* and *FCGR2C* were associated with Kawasaki Disease susceptibility and influenced IVIG treatment response (72). Furthermore, the *FCGR2B* minor alleles (IIB-386c and IIB-120a) conferring increased promoter activity were positively correlated to IVIG therapeutic response, although with limited statistical power over a small sample size (73). Each of these genetic associations is also constrained by unequal polymorphic variation between the different ethnic groups studied.

Altogether, particular FcγR polymorphisms have been described to be associated with the induction or severity of antibody-related disease, or patient responsiveness to antibody-based therapies. Nonetheless one should keep in mind that most FcγR-encoding genes are located within the 1q23 locus (*FCGR2A, FCGR3A, FCGR2B, FCGR2C, FCGR3B*) and may display a high degree of linkage disequilibrium, as reported for *FCGR2A* and *FCGR3A* (74) and for *FCGR2C* and *FCGR3B* (44). Association studies of FcγR-encoding genes should therefore include analyses of all FcγR-encoding genes from the 1q23 locus, and not focus on one particular gene.

## *In vivo* Roles of Human FcγRs: Lessons from Mouse Models^1^

### Transgenic mouse models expressing hFcγR(s)

Transgenic mouse studies have greatly enhanced our understanding of the *in vivo* function of hFcγRs. In particular, these studies have highlighted the respective contributions of hFcγRs to antibody-mediated inflammatory and allergic diseases (refer to Section “Understanding the Role of hFcγRs *In vivo* Using Transgenic Mouse Models: Illustrated in Autoimmune, Inflammatory, and Allergic Diseases”). Over the last two decades, various transgenic mouse strains have been generated that carry single or multiple hFcγR-encoding genes (Table 2). Transgenic strains were initially generated on a wild-type mouse background; however, later studies have examined transgene expression in mice deficient for multiple endogenous mFcγRs, to specifically study the function of the transgenic human receptor.

**Table 2:** **Association of FcγRs receptor variants with chronic inflammatory or immunological diseases**.

Gene	SNP	Disease	Reference
*FCGR2A*	H_131_	GBS, Kawasaki disease, idiopathic pulmonary fibrosis, and, for homozygous genotypes, MG, and children chronic ITP	(75–79)
	R_131_	Bronchial asthma and allergic rhinitis, Still disease, Behçet’s disease, refractory ITP, WG, MS, SLE, lupus nephritis, antiphospholipid syndrome, giant cell arteritis, rheumatic fever, ITP, and IgA nephropathy	(55, 60, 80–94)
	FcγRIIa-exon 6*	Anaphylaxis in patients with hypogammaglobulinemia, common variable immunodeficiency	(41)
*FCGR2B*	T_232_	SLE, anti-GBM disease	(57, 95–99).
	−386C/−120A	SLE, chronic inflammatory demyelinating polyneuropathy	(42, 100, 101)
*FCGR2C*	CNV	ITP, Kawasaki disease	(44, 72)
*FCGR3A*	F_158_	SLE, Crohn’s disease, Behçet’s disease, severe GBS, bullous pemphigoid, WG relapses, RA, and for homozygotes, chronic ITP, and nephritis	(45, 60, 67, 70, 77, 93, 102–105)
	V_158_	For homozygotes: RA susceptibility and severity, idiopathic inflammatory myopathies, and IgA nephropathy	(90, 106–108)
	CNV	Anti-GBM disease, RA	(109, 110)
*FCGR3B*	NA1	For homozygotes: anti-neutrophil cytoplasmic antigen systemic vasculitis, chronic ITP in children, and severe course of MG	(75, 77, 111, 112)
	NA2	SLE, severe GBS, Behçet’s disease, IgA nephropathy, and MS	(85, 93, 105, 111, 113)
	SH	Alloimmune neonatal neutropenia, transfusion reactions	(50)
	CNV	Glomerulonephritis, SLE, systemic autoimmunity, RA, idiopathic pulmonary fibrosis, systemic sclerosis, and Kawasaki disease	(52, 72, 114–118)

*GBM, glomerular basement membrane; GBS, Guillain–Barré syndrome; ITP, idiotypic thrombocytopenic purpura; MG, myasthenia gravis; MS, multiple sclerosis; RA, rheumatoid arthritis; SLE, systemic lupus erythematosis; SNP, single nuclear polymorphism; WG, Wegener’s granulomatosis*.

The common approach to reproduce hFcγR expression patterns in mice is to use the genuine human promoter to drive transgene expression (Table 2). Whereas this strategy was successful for hFcγRIIA^tg^ and hFcγRIIIB^tg^ mice, both hFcγRI^tg^ mice and hFcγRIIB^tg^ mice exhibit somewhat abnormal expression [discussed in Ref. (62)]. hFcγRI^tg^ mice, for example, constitutively express substantial amounts of this receptor on neutrophils (37), while in humans hFcγRI is only inducibly expressed on neutrophils in contexts of inflammation, infection and during particular therapies [reviewed in Ref. (62)]. An alternative strategy consists of using a cell-specific promoter to drive hFcγR expression. hFcγRIIA^tg^, hFcγRIIIB^tg^, or double-transgenic mice were generated using the human MRP8 promoter to express these receptors on neutrophils and, abnormally for hFcγRIIIB, on a proportion of monocytes (34). Finally, efforts made to cross the five single hFcγR-transgenic mouse strains with mFcγR^null^ mice – lacking mFcγRI, IIB, III, and IV – yielded a mouse model expressing most human IgG receptors – hFcγRI, IIA, IIB, IIIA, and IIIB – that preserves most human expression patterns (119) (Table 2).

### Understanding the role of hFcγRs *in vivo* using transgenic mouse models: Illustrated in autoimmune, inflammatory, and allergic diseases

FcR-mediated uptake of immune complexes and subsequent antigen presentation is a critical aspect of the immune response to foreign pathogens. Targeting of antigen to hFcγRI in hFcγRI^tg^ mice induced a strong antibody response, suggesting that hFcγRI on myeloid cells is capable of mediating antigen uptake and presentation *in vivo* (37, 120, 121). Various studies have demonstrated the capacity for hFcγRI and hFcγRIIIA to mediate cytotoxicity in the form of anti-tumor activity when engaged by bi-specific antibodies or antibodies with enhanced FcR binding, highlighting the effectiveness of such engineered antibody therapeutics *in vivo* (122–125). The role of FcγR in mediating anti-tumor therapies has recently been well-reviewed elsewhere (126, 127) and will not be discussed further in this review. hFcγR-transgenic mice have been useful both in understanding the *in vivo* function of these receptors and dissecting pathological mechanisms of disease; for illustration this section will describe results obtained in models of autoimmune thrombocytopenia, anaphylaxis, inflammation, and RA. Clearly, the biological responses to immobilized IgG are a function of their location, structure, and deposition, determining the subsequent recruitment and FcγR-mediated activation of immune cells: hFcγR-transgenic mice can assist us also in understanding the cell-specific role of FcγR in recruitment and immune complex clearance.

#### Autoimmune thrombocytopenia

Mice deficient for the FcRγ-subunit that is necessary for the expression of all mouse activating FcγRs are resistant to antibody-mediated platelet destruction, demonstrating the importance of activating FcγRs in this model of autoimmune thrombocytopenia (128). Using transgenic mice, both hFcγRI and hFcγRIIA were found to be independently sufficient for platelet clearance (9, 129). In hFcγRI^tg^ mice, thrombocytopenia was mediated by monocyte/macrophages outside of the spleen (9), whereas in hFcγRIIA^tg^ mice, splenectomy was found to provoke a more severe phenotype of thrombosis and systemic shock when thrombocytopenia was induced by activating anti-platelet antibodies (130). Importantly, hFcγRIIA is the only FcγR expressed on platelets, in humans and hFcγRIIA^tg^ mice. It is likely, therefore, that the presence of this FcγR on the platelets themselves contributes to antibody-induced intravascular platelet activation that is most efficiently resolved by phagocytes in the spleen. These findings have implications for understanding human immune-mediated thrombocytopenic disorders, such as heparin-induced thrombocytopenia/thrombosis (HIT/T), a serious complication arising from the clinical use of heparin. Using hFcγRIIA^tg^ mice it was identified that antibodies against heparin–platelet factor 4 complexes are responsible for hFcγRIIA-mediated platelet activation, thrombocytopenia, and thrombi formation in the lung vasculature (131, 132). Similarly, thromboembolic complications from the use of monoclonal antibody therapies may be a result of hFcγRIIA-dependent platelet activation due to circulating immune complexes (133, 134). Another important outcome of these mouse studies is that the density of hFcγRIIA expression in the transgenic animal affects the severity of antibody-induced disease (130), which has critical ramifications for understanding differences in immune reactions between individuals. Finally, a therapeutic intervention targeting the hFcγRIIA-signaling pathway proved successful for the prevention of thrombocytopenia in hFcγRIIA^tg^ mice (135).

#### Anaphylactic reactions

Individuals who have developed antibodies against a given allergen can, upon re-exposure, develop a severe systemic allergic reaction (anaphylaxis). Allergen re-exposure induces the rapid formation of immune complexes that leads to cellular activation and release of vasoactive mediators, which drives the phenotype of systemic shock, including symptoms of hypotension and respiratory distress. Although anaphylaxis is classically attributed to an IgE-mediated mast cell-dependent paradigm of allergic reactivity, the same systemic symptoms can be reproduced experimentally in mice by the transfer of specific IgG antibodies and allergen, of preformed immune complexes (passive systemic anaphylaxis, PSA), or by repeated immunization with an antigen prior to challenge (active systemic anaphylaxis, ASA). hFcγRI and hFcγRIIA expressed in transgenic mice were each individually sufficient to mediate PSA, the symptoms of which may be alleviated by pre-treatment with blocking antibodies (9, 136). PSA mediated by hFcγRIIA was found to be independent of mast cells and basophils, but rather dependent on neutrophils and monocytes/macrophages (136). Furthermore, hFcγRI and hFcγRIIA were identified as each individually sufficient to mediate ASA in transgenic mice, resulting in both hypothermia and death (9, 136). hFcγRI-dependent ASA required neutrophils and the release of platelet activating factor (9). These data demonstrate that hFcγR expressed on neutrophils and monocytes can mediate fatal anaphylactic reactions *in vivo*. Furthermore, in hFcγRI^tg^IIA^tg^IIB^tg^IIIA^tg^IIIB^tg^ mice (on the mFcγR^null^ background), administration of aggregated IgG was sufficient to trigger anaphylaxis (119). In addition, directly targeting either hFcγRI or hFcγRIIA by injection of agonistic mAb could induce anaphylaxis in transgenic mice (9, 136). Altogether, these data support the notion that anaphylaxis may also occur in humans in an hFcγR-dependent manner when allergen-specific IgGs are produced by an individual.

#### Immune complex induced inflammation

The formation of immune complexes is a hallmark of many human diseases, and their accumulation is an important trigger of inflammation-induced tissue damage. Pathogenic antibodies may bind directly to host cells, or immune complexes may deposit within tissues and trigger activation of local or circulating hFcγR-expressing cells. Using hFcγRIIA^tg^ mice, it was demonstrated that hFcγRIIA expressed on skin mast cells could trigger their activation following intradermal injection of immune complexes resulting in an inflammatory reaction in the skin (136). Inflammation of the airways due to local formation of immune complexes is characterized by granulocyte infiltration, elevated levels of myeloperoxidase, and subsequent damage to the lung epithelium, mimicking symptoms of asthmatic disease in humans. Whereas FcRγ-subunit^−/−^ mice are resistant to IC-induced airway inflammation, transgenic expression of either hFcγRI or hFcγRIIA was sufficient to restore this antibody-mediated pathology (9, 136).

#### Rheumatoid arthritis

Rheumatoid arthritis is an autoimmune disease in which the formation of immune complexes within the joints drives an inflammatory pathology. Autoantibodies directed against joint proteins such as collagen type II or glucose-6-phosphate isomerase (GPI) are found in RA patients, and the arthritis pathology may be modeled in mice by either active immunization with joint-associated components or by passive antibody transfer. hFcRn^tg^ mice provided direct evidence for the role of this receptor in serum persistence and transport of antibodies into tissues (23). Indeed, mFcRn^−/−^ mice are resistant to passive arthritis induction, and transgenic expression of hFcRn could restore arthritis susceptibility (137, 138); suggesting that greater IgG serum persistence may have implications for many autoimmune and inflammatory conditions (139). Surprisingly, transgenic expression of hFcγRIIA-R_131_ on a wild-type mouse background was associated with the spontaneous development of an RA-like joint pathology (140). Expression of hFcγRIIA indeed renders mice highly susceptible to various models of arthritis (140, 141), even if its expression is purposely restricted to neutrophils (142). Small inhibitors designed to bind antagonistically to hFcγRIIA were found to be protective (143), proposing a hFcγR-targeted therapy for RA. Besides hFcγRIIA^tg^ mice, other hFcγR-transgenic mice do not exhibit spontaneous joint inflammation. Nevertheless, hFcγRI^tg^ mice demonstrated that this receptor is sufficient to mediate arthritis induction in transgenic mice, dependent on the presence of both neutrophils and monocytes/macrophages (9). Therapeutic elimination of inflammatory macrophages by an hFcγRI-targeting immunotoxin inhibited the progression of experimental arthritis in hFcγRI^tg^ rats (144), and resolved cutaneous inflammation (145).

#### Cell-specific function of FcγR

Studies using hFcγR^tg^ mice have enabled the description of specific *in vivo* functions not only for these IgG receptors, but also the cells that express them. Neutrophils are a particularly relevant example: the two main human neutrophil IgG receptors, hFcγRIIA and hFcγRIIIB, were found to individually and cooperatively promote IC-induced neutrophil recruitment and accumulation in the tissues. hFcγRIIA alone, however, promoted associated injury and inflammation in multiple models of antibody-dependent autoimmunity. Importantly, neutrophil recruitment occurred despite the absence of FcγR expression on other cell types such as mast cells and macrophages, indicating a prominent role for hFcγRs on neutrophils in IC-induced recruitment (34). Furthermore, specialized functions may be attributed to these two neutrophil FcγR: hFcγRIIIB seems to play an important role in homeostatic clearance of immune complexes deposited within the vasculature, whereas in a complex environment of immune complex deposition within the tissue and the vasculature, hFcγRIIA was required for the formation of neutrophil extracellular traps (NETs) (146). Collectively, these data in hFcγR^tg^ mice demonstrate the value of a transgenic approach to appreciate the role of human FcγR and the cells expressing them.

## Final Considerations

Although, it is tempting to draw conclusions from genetic association studies performed in humans, it would be overreaching to delineate causal relationships between particular FcγR variants and antibody-mediated human disease. Importantly, all the human FcγR-transgenic mouse strains that have been reported express a single polymorphic variant of each FcγR (Table 3). Thus, no comprehensive study can compare today the properties of a given polymorphism in mouse models of disease. Novel mouse models based on the exchange of the entire FCGR locus with that of humans may allow these comparison studies, or transgenic/knock-in mice expressing different polymorphic variants than the transgenic mice already reported, but remain to be generated. Still, when taking into account published data from both humans and animal models (referenced in Tables 2 and 3) several parallel observations have been described:

- Expression of hFcγRIIA (R_131_) renders mice susceptible to arthritis and autoimmune pathologies including thrombocytopenia (Table 3); and expression of hFcγRIIA-R_131_ allotype is similarly associated with inflammatory diseases, thrombocytopenia, and autoimmunity in humans (Table 2). The FcγRIIa-exon 6* polymorphic variant, which confers increased neutrophil sensitivity to IgG stimulation (Table 1) was also associated with anaphylactic responses in patients upon IVIG therapy (Table 2); consistent with data obtained in hFcγRIIA^tg^ mice indicating that neutrophils can contribute to IgG-dependant anaphylaxis mediated by FcγRIIA.
- The NA1 allotypic variant of FcγRIIIB confers increased phagocytosis of IgG-immune complexes, and is associated with thrombocytopenia in humans; whereas FcγRIIIB-NA2 and CNV are associated with inflammatory and autoimmune conditions characterized by immune complex deposition. These data are congruent with findings in NA2-hFcγRIIIB^tg^ mice (Table 2), demonstrating an important role for this receptor in mediating neutrophil recruitment as well as homeostatic clearance of immune complexes.

**Table 3:** **hFcγR-transgenic mouse models: description and main results obtained**.

Promoter	Expression	Variant	Strain	*In vivo* findings	Reference
**CD64 (hFcγRI)**					
*FCGR1*	Monocytes, macrophages, DCs, neutrophils		FVB/N	Bi-specific mAb-dependent hFcγRI-triggered killing (*in vitro*)	(122)
			FVB/N	Anti-hFcγRI mAb immunization elicits higher Ab responses	(37)
			FVB/N	hFcγRI-mediated binding and phagocytosis of opsonized RBCs	(147)
			?	Antigen targeting to hFcγRI increased vaccination potency	(120)
			FVB/N	Weak antigen targeting to hFcγRI enhances immunogenicity	(121)
			FVB/N	Immunotoxin targeting of hFcγRI reduces inflammation	(145)
			5KO (B6 F6)	hFcγRI-dependent arthritis, thrombocytopenia, airway inflammation, and anaphylaxis (PSA and ASA)	(9)
**CD32A (hFcγRIIA)**					
*FCGR2A*	Monocytes, macrophages, neutrophils, eosinophils, basophils, mast cells, DCs, megakaryocyte, platelets	R_131_	FcRγ^−/−^(B6xSJL)	Immune thrombocytopenia can be induced via hFcγRIIA	(129)
			FcRγ^−/−^(B6)	hFcγRIIA-dependent thrombosis and shock	(130)
			hPF4^tg^ (B6)	hFcγRIIA-dependent Heparin-induced thrombocytopenia	(131)
			C57BL/6	Increased active and passive collagen-induced arthritis	(140)
			FcRγ^−/−^(B6xSJL)	hFcγRIIA mediates experimental immune hemolytic anemia	(148)
			hPF4^tg^ lo/hi (B6)	PF4-hFcγRIIA-dependent Heparin-induced thrombocytopenia	(132)
			C57BL/6 × SJL F_1_	hFcγRIIA-dependent platelet activation by Bevacizumab IC	(133)
			C57BL/6 × SJL F_1_	Small chemical entities inhibit collagen-induced arthritis	(143)
			C57BL/6 × SJL F_1_	hFcγRIIA-dependent platelet activation by CD40L IC	(134)
			C57BL/6 × SJL F_1_	Increased sensitivity to autoimmune arthritis	(141)
			C57BL/6	Inhibition of hFcγRIIA-signaling pathway to inhibit thrombosis and thrombocytopenia	(135)
			FcRγ^−/−^,5KO	hFcγRIIA induces anaphylaxis and airway inflammation	(136)
			C57BL/6J	hFcγRIIA cooperates with integrin signaling in platelets	(149)
*MRP8*	Neutrophils, some monocytes	R_131_	FcγR^−/−^	hFcγRIIA-dependent nephritis, Arthus reaction, neutrophil recruitment and tissue injury	(34)
			FcγR^−/−^	Neutrophil hFcγRIIA is sufficient for arthritis induction	(142)
			FcγR^−/−^	hFcγRIIA-dependent NETosis in Arthus reaction	(146)
**CD32B (hFcγRIIB)**					
*FCGR2B*	B cells, splenic CD11c DCs, monocytes, neutrophils, eosinophils	I_232_	C57Bl/6	Crosslinking hFcγRIIB and CD19 suppresses humoral immunity in systemic lupus erythematosus	(150)
			FcRγ^−/−^or FcγRIIB^−/−^	hFcγRIIB-enhanced immunostimulatory and anti-tumor activity of chimeric mouse–human agonistic anti-CD40 Abs	(151)
			CD40^−/−^	Anti-tumor activity of agonistic anti-TNFR Abs requires differential hFcγRIIB coengagement	(152)
**CD16A (hFcγRIIIA)**					
*FCGR3A*	NK cells, macrophages	F_158_	B6xCBAFl	Promoter/expression analysis	(153)
?	NK cells and ?	?	SCID	Glycoengineering of a humanized anti-EGFR Ab leads to enhanced ADCC through hFcγRIIIA	(125)
**CD16B (hFcγRIIIB)**					
*FCGR3B*	Neutrophils	?	B6xCBAFl	Promoter/expression analysis	(153)
*MRP8*	Neutrophils, some monocytes	NA2	FcRγ^−/−^	hFcγRIIIB is sufficient for NTS nephritis, cutaneous RPA reaction and promotes neutrophil recruitment	(34)
			FcRγ^−/−^	hFcγRIIIB mediates neutrophil tethering to intravascular immune complexes and their uptake	(146)
**CD32A (hFcγRIIA) + CD16B (hFcγRIIIB)**					
*MRP8*	Neutrophils, some monocytes	IIA: R_131_	FcRγ^−/−^	hFcγRIIA and hFcγRIIIB cooperate to induce nephritis and cutaneous Arthus reaction	(34)
		IIIB:NA2	
**FcγR-HUMANIZED MICE (INTERCROSS OF hFcγRI^tg^, IIA^tg^, IIB^tg^, IIIA^tg^ AND IIIB^tg^ MICE)**					
*FCGR1*	Please refer to single transgenic mice	I	mFcγRI^−/−^	Antibody-mediated FcγR-dependent cell depletion (B cells, T cells, platelets), and B16-F10 lung metastasis clearanceFcγR-mediated IC-induced systemic anaphylaxis	(119)
*FCGR2A*		IIA-R_131_	mFcγRIIB^−/−^		
*FCGR2B*		IIB-I_232_	mFcγRIII^−/−^		
*FCGR3A*		IIIA-F_158_	mFcγRIV^−/−^		
*FCGR3B*		IIIB-?			
**hFcRn**					
*FCGRT*	Intestine and ?		mFcRn^−/−^	hFcRn expression restores serum half life of hIgG in mFcRn^−/−^mice	(154)
			mFcRn^−/−^; mFcRn^−/−^FcγRIIB^−/−^	hIgG with engineered high FcRn binding affinity has enhanced half life *in vivo*; inhibition of the binding of pathogenic Abs to hFcRn ameliorates arthritis	(137)
			mFcRn^−/−^mβ2m^−/−^hFcRn^tg^ hβ2m^tg^	Blocking hFcRn using a peptide antagonist increases hIgG catabolism	(155)
			6KO (B6 F6)	hFcRn restores arthritis susceptibility in 6KO mice	(138)

*?, information unavailable in the original publication*.

While genetic association studies identify important risk factors and inform on the involvement of FcγR in human disease; hFcγR^tg^ mice allow us to more precisely dissect pathological mechanisms, and describe the role of human FcγR and the cells expressing them in various clinically relevant pathologies. Together, these data in humans and transgenic models highlight the contribution of hFcγR to antibody-mediated diseases, and open avenues for understanding pathogenic mechanisms. Such data will continue to impact on therapeutic choices and potentially identify new interventional targets.

## Conflict of Interest Statement

The authors declare that the research was conducted in the absence of any commercial or financial relationships that could be construed as a potential conflict of interest.

## Footnotes

^1^Note: for the sake of clarity, this section will use the terminology “hFcγR” for human IgG receptors, and “mFcγR” for mouse IgG receptors.
